# Comparative Study of Physicochemical Properties of Biochar Samples Derived from Nutshells as a Solid Fuel for Direct Carbon Solid Oxide Fuel Cells

**DOI:** 10.3390/ma18092112

**Published:** 2025-05-04

**Authors:** Magdalena Dudek, Bartosz Adamczyk, Anita Zych, Katarzyna Król, Przemysław Grzywacz, Krystian Sokołowski, Krzysztof Mech, Maciej Sitarz, Piotr Jeleń, Magdalena Ziąbka, Maja Mroczkowska-Szerszeń, Małgorzata Witkowska, Joanna Kowalska

**Affiliations:** 1 Faculty of Energy and Fuels, AGH University of Krakow, Mickiewicza 30 Av., 30-059 Cracow, Poland; badamczyk@agh.edu.pl (B.A.); zycha@agh.edu.pl (A.Z.); kasiakro@student.agh.edu.pl (K.K.); grzywacz@agh.edu.pl (P.G.); 2 Academic Centre for Materials and Nanotechnology, AGH University of Krakow, Mickiewicza 30 Av., 30-095 Cracow, Poland; krysok@agh.edu.pl (K.S.); kmech@agh.edu.pl (K.M.); 3 Faculty of Materials Science and Ceramics, AGH University of Krakow, Mickiewicza 30 Av., 30-059 Cracow, Poland; msitarz@agh.edu.pl (M.S.); pjelen@agh.edu.pl (P.J.); ziabka@agh.edu.pl (M.Z.); 4 Oil and Gas Institute—National Research Institute, Lubicz 25a, 30-350 Cracow, Poland; mroczkowska-szerszen@inig.pl; 5 Faculty of Metals Engineering and Industrial Computer Science, AGH University of Krakow, Mickiewicza 30 Av., 30-059 Cracow, Poland; witkowsk@agh.edu.pl (M.W.); joannak@agh.edu.pl (J.K.)

**Keywords:** biochar, direct carbon fuel cell, pyrolysis, walnut shells

## Abstract

This paper presents the results of an investigation into the effect of the physicochemical properties of carbon chars (biochars) on the performance of direct carbon solid oxide fuel cells (DC-SOFCs). Biochars were obtained from walnut, coconut, pistachio, hazelnut and peanut shells by pyrolysis at a temperature of 850 °C. The results of structural studies conducted using X-ray diffraction and Raman spectroscopy reflected a low degree of graphitisation of carbon particles. Biochar derived from walnut shells is characterised by a relatively uniform content of alkali elements, such as sodium, potassium, calcium, magnesium and iron, which are natural components of the mineral residue and act as catalysts for the Boudouard reaction. This study of gasification of biochar samples in a CO_2_ atmosphere recorded that the highest conversion rate from solid phase to gaseous phase was for the biochar sample produced from walnut shells. The superior properties of this sample are directly connected to structural features, as well as to the random distribution of alkali elements. DC-SOFCs involving 10 mol% of Sc_2_O_3_, 1 mol% of CeO_2_, 89 mol% of ZrO_2_ (10S1CeZ) or 8 mol% of Y_2_O_3_ in ZrO_2_ (8YSZ) were used as both solid oxide electrolytes and components of the anode electrode. It was found that the highest electrochemical power output (P_max_) was achieved for DC-SOFCs fuelled by biochar from walnut shells, with around 103 mW/cm^2^ obtained for such DC-SOFCs involving 10S1CeZ electrolytes.

## 1. Introduction

Solid oxide fuel cells (SOFCs) fuelled by solid carbon-rich waste materials continue to receive considerable attention. Direct carbon solid oxide fuel cells (DC-SOFCs) offer several advantages over hydrogen–oxygen SOFCs: (i) they do not require pre-reforming of the fuel; (ii) the risk of explosion is significantly lower than in hydrogen fuel cells and (iii) storing solid fuels is simpler than storing hydrogen. High-purity synthetic carbon, lignite, hard coal, coke, charcoal and carbon-rich fuels derived from various waste materials can all be used in DC-SOFCs. However, the optimal fuels for these fuel cells have not yet been identified [1,2,3].

In DC-SOFCs, carbon particles can be directly electrochemically oxidised as follows:

C + 2O^2−^ → CO_2_ + 4e− (1)

and through a sequence of electrochemical reactions:

C + O^2−^ → CO + 2e− (2)

CO + O^2−^ → CO_2_ + 2e−. (3)

In these DC-SOFCs, the solid carbon fuel is placed on the anode electrode in the fuel chamber, either in the form of pulverised powder or pressed carbon samples [4,5]. The chemical reactions (1)–(3) describe the operation of DC-SOFCs fuelled with high-purity powdered solid fuel.

One of the notable categories of solid fuels that can be applied in DC-SOFCs is organic waste materials. These are used as carbonaceous solid fuels for DC-SOFCs and are typically characterised by a high proportion of volatile components [6,7]. Solid carbon fuels produced from wood waste, agricultural and food industry waste, cardboard, paper and materials from the furniture industry have all been used to power DC-SOFCs. The high operating temperature of DC fuel cells facilitates the formation of small carbon particles through the direct pyrolysis of biomass within the cell [8,9,10,11].

The Boudouard reaction, which can be described as follows:

C + CO_2_ → 2CO (4)

involving CO_2_ and carbon as reactants, may serve as an additional source of CO consumed in reaction (3). The high operating temperatures (600–900 °C) during DC-SOFC operation favour Boudouard gasification of carbon, with CO_2_ acting as a major gasification agent. CO_2_ is a natural by-product of electrochemical reactions (1)–(3) in DC-SOFC operation. The concentration of CO_2_ in the anode chamber is sufficiently high for the gas to spontaneously isolate solid carbon particles within the carbon bed.

In DC-SOFCs supplied with biochar particles, both the electrochemical oxidation of carbon particles as solid fuel and the oxidation of CO as an intermediate fuel must be considered. This suggests that the presence of CO in the anode chamber is crucial for DC-SOFC performance [12,13,14]. The mineral residue, an integral part of the organic starting material, serves as a valuable catalyst in the Boudouard reaction. The literature data indicate that DC-SOFCs fuelled by various biochars derived from solid waste can achieve an electrical power output (Pmax) in the range of around 50–320 mW/cm^2^ [15,16,17,18].

Cai et al. [19] demonstrated the possibility of applying biocarbon derived from orchid tree leaves to supply DC-SOFC single cells. It was reported that the performance of DC-SOFCs supplied by leaf chars exceeded 210 mW/cm^2^ at a temperature of 850 °C. The naturally accumulated and uniformly distributed elements Ca and Mg in the leaves act as catalysts in the biochar during the reverse Boudouard reaction in the anode chamber of the DC-SOFC.

Q. Qiu et al. [20] described an analysis of results from DC-SOFCs supplied by chars derived from wheat straw, corn cobs or bagasse. The obtained maximum electrical power varied between approximately 187 mW/cm^2^ and 260 mW/cm^2^ at 800 °C. The long-term performance of such fuel cells was also observed. A DC-SOFC supplied with 0.5 g of char operated under a constant current load of 140 mA/cm^2^ for a duration of 15–24 h. The correlation between physicochemical properties, specifically the content of elements such as K, Si, S, Ca, Mg and Fe in the fuel, and their influence on performance in the DC-SOFC was also noted.

H. Wou [21] studied the in situ catalytic gasification of kelp-derived biochar as a fuel for DC-SOFCs. The utilisation of this type of fuel allowed for a maximum electrical power output of approximately 285 mW/cm^2^ at 850 °C.

T. Chen et al. [22] studied the impact of the preparation of Camellia oleifera biomass on the performance of DC-SOFCs. Both the raw solid fuel and the char obtained from Camellia oleifera were tested in DC-SOFCs. The application of both carbonaceous materials resulted in electrical power outputs exceeding 300 mW/cm^2^ at a temperature of 800 °C.

These results clearly indicate the potential application of new types of fuel for DC-SOFCs derived from waste. The most important and common feature is the presence of naturally accumulated inorganic elements in the cell walls and their impact on the chemical reactivity of such chars in the reverse Boudouard reaction, which also has a considerable effect on the resulting current density and electrical power of the DC-SOFC.

Although very promising results regarding the electrochemical performance of DC-SOFCs have been obtained for different types of biochars derived from various waste materials, there is a lack of systematic research examining the influence of the type of raw carbonaceous waste material on the physicochemical properties of biochar, and consequently, on its performance as a fuel under DC-SOFC operating conditions [23,24].

There are also various reasons for the disparity among results regarding the operating parameters of SOFCs, including:

(i) different degrees of graphitisation of the carbon materials used as fuel
(ii) variations in total content, as well as differences in the qualitative and quantitative composition of individual inorganic compounds within the mineral matter that act as natural catalysts for the Boudouard reaction
(iii) features characterising the carbon particles, such as morphology, particle size distribution, surface area, total porosity in solid fuels and porosity size distribution [25,26,27].

Further differences may result from the varying chemical compositions of components used in solid oxide fuel cells, such as cathode materials, oxide electrolytes and anode materials. Additionally, different DC-SOFC configurations are employed to investigate electrical power output. The impact of experimental conditions during DC-SOFC testing has also been a crucial factor in determining the obtained current and power density [28,29,30].

In previous studies [31,32], we have reported the effect of pyrolysis temperature on the physicochemical properties of biochars derived from walnut and pistachio shells. We also analysed the differences in the operating parameters of DC-SOFCs supplied with these fuels. Based on these studies, it was found that biochar samples obtained at temperatures above 400 °C exhibit high electrochemical reactivity in DC-SOFCs due to the presence of highly defective, non-ordered carbon particles. It was further observed that pyrolytic products evolved from carbon powders also influence the resulting current and power density of DC-SOFCs.

The subject of the experimental investigations described in this article is fully charred biochar samples obtained from nutshells. The properties of these chars differ from those of natural feedstock or carbon samples with a lower degree of carbonisation. These samples possess a higher level of moisture and volatile components. In contrast, chars subjected to higher carbonisation exhibit significantly higher carbon content and lower hydrogen content. Furthermore, the removal of volatiles leads to an increased ash content, which affects the alkali index. These experimental factors are crucial for tailoring the physicochemical properties of biochars and, in turn, their impact on the kinetics and mechanism of electrochemical oxidation of carbon particles in DC-SOFCs.

The aim of this study is to investigate the physicochemical properties of fully carbonised biochar samples derived from nutshells and to demonstrate their impact on the performance of DC-SOFCs. Thus, it will be possible to select an optimised fuel within this group, as well as determine the preferred SOFC construction.

## 2. Materials and Methods

### 2.1. Description of Sample Selection and Biochar Preparation

All biochar samples were prepared via the thermal conversion of ground walnut, coconut, hazelnut, pistachio and peanut shells at a temperature of 850 °C for 1 h in a laboratory flow reactor. Small portions of each respective feedstock were subjected to thermal conversion at 850 °C under a nitrogen (N_2_) flow rate of 50 mL/min. Previous papers have documented the scheme and details of the laboratory flow reactor and described the procedure applied for the thermal conversion of all feedstocks used in preparing the investigated biochars [33].

The abbreviations introduced for the biochar samples or raw shells obtained from the individual sources of waste biomass are used consistently throughout the study.

### 2.2. Analytical Methods for the Evaluation of the Physicochemical Properties of Biochars

The fully charred samples listed in Table 1 were subjected to primary elemental analysis for carbon, sulphur and hydrogen content. Biochar was analysed using the ELTRA CHS-580 chemical analyser (ELTRA GmbH, Haan, Germany). Humidity and ash content were determined through gravimetric analysis.

The X-ray diffraction (XRD) method was employed to assess the phase composition and degree of disorder in the biochar samples. Diffraction analysis was conducted using a Siemens D500 diffractometer with a filtered lamp featuring a copper anode (λ = 0.15418 nm) and the step count method (∆2θ = 0.04°, counting time τ = 10 s/step, angle range 2θ = 10–120°). XRD analysis was also used to determine the phase composition of ash derived from biochar samples. Measurements were performed both before and after electrochemical testing.

Raman spectroscopy was utilised to examine the specific structural properties of biochar as a solid fuel. Raman spectra were recorded using a confocal Raman spectrometer, LabRAM HR 800 (HORIBA SAS, Longjumeau, France), equipped with a 532-nm laser.

The Thermo Nicolet FTIR 6700 spectrometer was used to analyse biochar samples in the mid-infrared range (MID-IR), a fingerprint region for most organic and inorganic molecules. The system was equipped with a DLaTGS detector and an ETC Ever-Glo IR source on the XT-KBr beam splitters. IR spectra were obtained using the Specac^®^ Quest diamond ATR attachment with 128 repetitions. In this configuration, the system recorded spectra within the range of 400–4000 cm^−1^.

X-ray photoelectron spectroscopy (XPS) analyses were conducted using a PHI Versa Probe II Scanning XPS system with monochromatic Al Kα (1486.6 eV) X-rays focused on a 100-µm spot. The photoelectron take-off angle was 45°, and the pass energy in the analyser was set to 117.50 eV (0.5-eV step) for survey scans and 46.95 eV (0.1-eV step) for high-resolution spectra of C 1s, O 1s, N 1s and Ca 2p. A dual-beam charge compensation system, consisting of 7-eV argon⁺ ions and 1-eV electrons, was used to maintain a constant sample surface potential, regardless of sample conductivity. All XPS spectra were charge-referenced to the unfunctionalised saturated carbon (C-C) C 1s peak at 285.0 eV. The operating pressure in the analytical chamber was maintained below 3 × 10^−9^ mbar. Spectrum deconvolution was performed using PHI MultiPak software (v.9.9.3), and the spectrum background was subtracted using the Shirley method.

Scanning electron microscopy (SEM) was used to examine differences in the morphological features of carbon particles and their chemical composition in biochar powders obtained from different nutshells. The SEM system used was an Apreo 2S Low Vac SEM (Thermo Fisher Scientific, Waltham, MA, USA) with APEX™ Advanced 2022 software, version 2.5.1001.001 for energy-dispersive X-ray spectroscopy (EDX) (EDAX, Eindhoven, the Netherlands). Observations were conducted under high vacuum conditions using an ETD detector.

Microstructural observation and chemical analysis of all ceramic fuel cell components were also performed using SEM with the above-mentioned devices. The investigation was conducted on cross-sections prepared from complete SOFCs. The preparation procedure for SEM characterisation included epoxy impregnation and polishing.

The structural analysis of porous biochars was conducted using low-temperature (77 K) N_2_ adsorption isotherms with a 3FLEX fully automated, three-station instrument (Micromeritics Instrument Corporation, Norcross, GA, USA). These measurements were performed as a function of N_2_ pressure across a wide range of relative pressures. The experimentally determined N_2_ adsorption isotherms allowed for the characterisation of key biochar structural parameters, including specific surface area, pore volume and the pore volume distribution function.

The chemical analysis of inorganic elements in biochar used as fuel was performed using wavelength dispersive X-ray fluorescence (XRF) spectroscopy with an Axios MAX spectrometer (PANalytical, Malvern, UK). The system operates with a 4-kW rhodium tube equipped with a 30-μm-thick window, a maximum accelerating voltage of 60 kV and a maximum current of 150 mA. Qualitative spectrum analysis was conducted by identifying spectral lines, while quantitative analysis was performed using the fundamental parameters method for elements ranging from fluorine to uranium (F–U). The determined element contents were normalised to 100% by mass.

### 2.3. Thermal Behaviour Analysis of Biochars Obtained from Hard Nutshells

Thermal effects during the heating of solid carbon fuel within a temperature range of 25–1000 °C in an N_2_ gas stream were measured using the differential thermal analysis (DTA), differential scanning calorimetry (DSC) and thermo-gravimetric (TG) methods (Simultaneous Thermal Analyser 449 F3 Jupiter^®^). Samples (~50 mg) were heated at a rate of 10 °C/min in a platinum crucible. The measurements were conducted in an N_2_ atmosphere.

Thermogravimetry was also used to determine the chemical reactivity of the obtained charcoal samples with CO_2_ within a temperature range of 20–850 °C. A thermobalance (Rubotherm DynTHERM 1100-40 MP-G Analyser, TA Instruments, Bochum, Germany) was used for investigations in a CO_2_ gas atmosphere. The variation in mass loss versus temperature or time was recorded within a temperature range of 25–1100 °C in pure CO_2_ at a pressure of 0.1 MPa (abs). The gas flow rate was 100 mL/min, and the temperature ramp was 10 °C/min. The final heating temperature of 1100 °C was maintained for 20 min.

### 2.4. Electrochemical Investigations of the Performance of Direct Carbon Fuel Cells

The direct oxidation of carbon was studied using two types of electrochemical cells:

DC-SOFC (I): C|Ni-10Sc1CeSZ|10Sc1CeSZ|LSM-GDC|LSM

DC-SOFC (II): C|Ni-YSZ|8YSZ LSM-GDC|LSM

At present, it is common in DC-SOFC fuel cell research to use components frequently applied in the construction of hydrogen–oxygen SOFCs, since the target electrolytic or electrode materials have not yet been identified. Based on the analysis of research data collected globally and the evaluation of technical specifications, it appears that SOFCs constructed on either electrolyte or anode support, but containing 10 mol% Sc_2_O_3_—1 mol% CeO_2_—89 mol% ZrO_2_ (10 Sc1CeZ) solid solutions, as ceramic oxide ion conductors achieve higher electrical performance compared to SOFCs that typically use 8 mol% Y_2_O_3_ in ZrO_2_ (8YSZ) as an electrolyte.

Solid oxide fuel cells (SOFCs) containing 10Sc1CeSZ as the electrolyte with nickel particles distributed in 10Sc1CeSZ as the anode were selected for this study. The 10Sc1CeSZ electrolyte has higher ionic conductivity than the currently used 8YSZ, and a nickel–zirconium cermet (Ni-10Sc1CeSZ) has greater activity in the CO’s anodic oxidation in SOFCs compared to the commonly used Ni-YSZ [34,35]. The electrolyte-supported SOFCs were provided by the company KERAFOL^®^ Keramische Folien GmbH & Co KG (Koppe-Platz 1, Eschenbach in der Oberpfalz, Germany). Small, normalised disc-shaped ceramic fuel cells were used. The active surface area of both electrode materials was ~2 cm^2^. Names of the components used for the construction of direct carbon (DC)-SOFC chemical composition are listed in Table 2.

In this paper, the authors address the extent to which the selection of electrode and electrolytic materials with better properties than those commonly used will improve the performance of DC-SOFCs. This research is necessary as a parallel line of experimental efforts alongside studies on the optimisation of the physicochemical properties of biochar.

**Figure 1:**
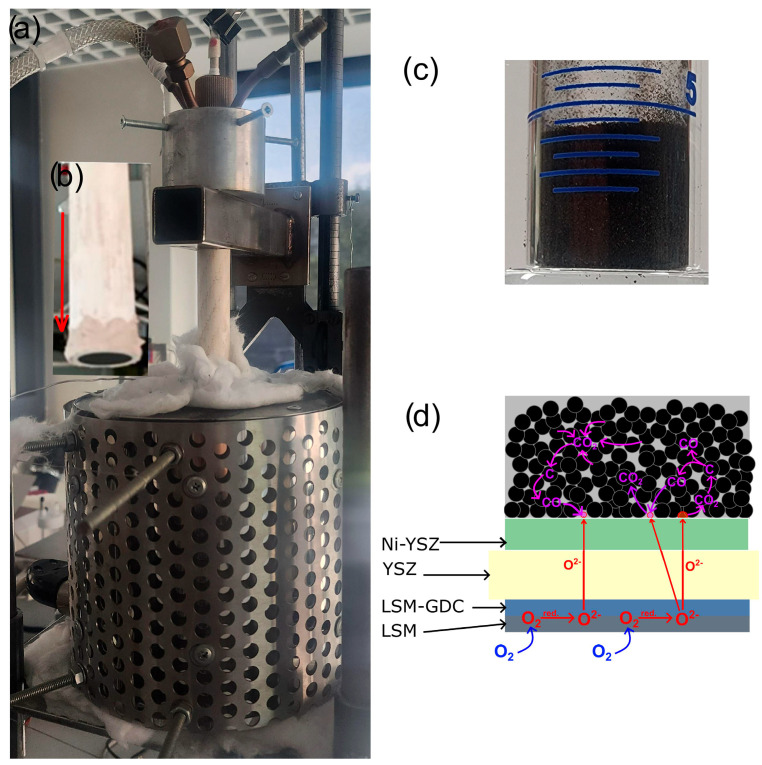
Photo of the DC-SOFC laboratory set-up for electrochemical investigations (**a**); disc-shaped ceramic fuel cells (DC-SOFC I or DC-SOFC II) bonded to an Al_2_O_3_ tube (**b**); biochar fuel placed on the top anode material Ni-10Sc1CeSZ or Ni-YSZ (**c**); a concept for the electrochemical performance of DC-SOFC fueled by solid carbon fuel (**d**).

Figure 1 presents a photograph of the DC-SOFC laboratory setup used for the electrochemical investigations and the concept behind DC-SOFC’s electrochemical performance supplied through solid carbon fuel.

The disc-shaped ceramic fuel cells are pasted to alumina tubes by high temperature ceramic glue. The biochar fuel is placed on top of the surface anode material Ni-10Sc1CeSZ or Ni-YSZ. Here, electrochemical measurements were performed within 700–850 °C using an Autolab workstation with a PGSTAT 300N potentiostat equipped with GPES (chrono and pulse techniques) and FRA (electrochemical impedance spectroscopy) modules. The current (I)—voltage (V) dependence of DC-SOFC cell (I) or DC-SOFC (II) were measured via cyclic voltammetry, which was conducted at a scan rate of 0.01 V/s. The registered dependencies voltage U (V)—current I (A) was used to calculate the current density j (A/cm^2^) and power density P (mW/cm^2^). The relationships of electrical dependencies U-j and P-j are presented in all graphs. The chronoamperometry technique (constant voltage mode) was used to determine the variations of current I vs. time (t) under polarisation (V). GPES software, which was provided by the supplier of the electrochemical workstation Autolab N-220, was used for the visualisation of the measured data. The experimental setup and methodology for electrochemical investigations are also described in previous papers [36].

## 3. Results

The basic elements characterizing the physicochemical properties of biochars include the total content of carbon, hydrogen, sulphur, and mineral residues relative to the mass proportion of the pure carbon fraction. Table 3 summarizes the fundamental features of the biochar samples obtained, including the results of chemical element composition analyses and ash content in the prepared biochar samples. This table also presents the analytical results for the same elemental compounds—carbon, hydrogen, and sulphur—in the starting raw materials.

The analysis of the disparate results regarding total carbon content showed that biochar obtained from coconut, hazelnut and walnut hard shells had a carbon content ranging from 88% to 96% in samples prepared under the same conditions.

The results of the analytical assessment of carbon, hydrogen, and sulfur content are in good agreement with data available in the literature for both the raw shells used as feedstock and the raw materials or biochars obtained from the selected nut shells [37,38].

These values are sufficient for the use of such biochars as solid fuels in DC-SOFCs. The samples were characterized by a negligible proportion of sulfur, below <0.01%. The biochar obtained from raw peanut shells exhibited a significantly lower carbon content (<88%) and a relatively high sulfur content (0.11%). This sulfur level may be a limiting factor when using this biochar as a fuel for DC-SOFCs.

Another important factor is the mineral residue content. The total amount of inorganic minerals, their qualitative composition, and the proportions of individual components are key factors influencing the electrochemical and chemical activity of solid fuel. The data presented in Table 3 also reflect the varying percentages of ash content [%], determined by the gravimetric method, for the biochar samples under investigation. Based on Table 3, biochar obtained at 850 °C from hard nutshells has an ash content ranging from approximately 1% to 2%. In contrast, the ash content of biochar derived from peanut shells was substantially higher, at around 5%.

In combustion technologies that rely on solid fuel, a high ash content is not beneficial, as it acts as ballast and reduces the calorific value of the fuel. In the case of biochars used as solid fuels in DC-SOFCs, determining the exact threshold at which mineral residue content negatively impacts the electrochemical performance of the cells is challenging. This complexity arises from the more intricate oxidation mechanism of solid fuels compared to gaseous fuels.

In DC-SOFCs, systematic research on the tolerable content of mineral residues in solid fuels remains limited. The role of individual inorganic compounds, which may act as catalysts in gasification processes while also increasing the internal electrical resistance of DC-SOFCs, is not yet fully understood.

The phase composition and the presence of additional phases identifying inorganic compounds in biochar samples are also key factors in assessing the suitability of biochar as a solid fuel for DC-SOFCs. Figure 1a presents the XRD pattern of a series of biochars obtained from walnut, pistachio and hazelnut hard shells.

**Figure 2:**
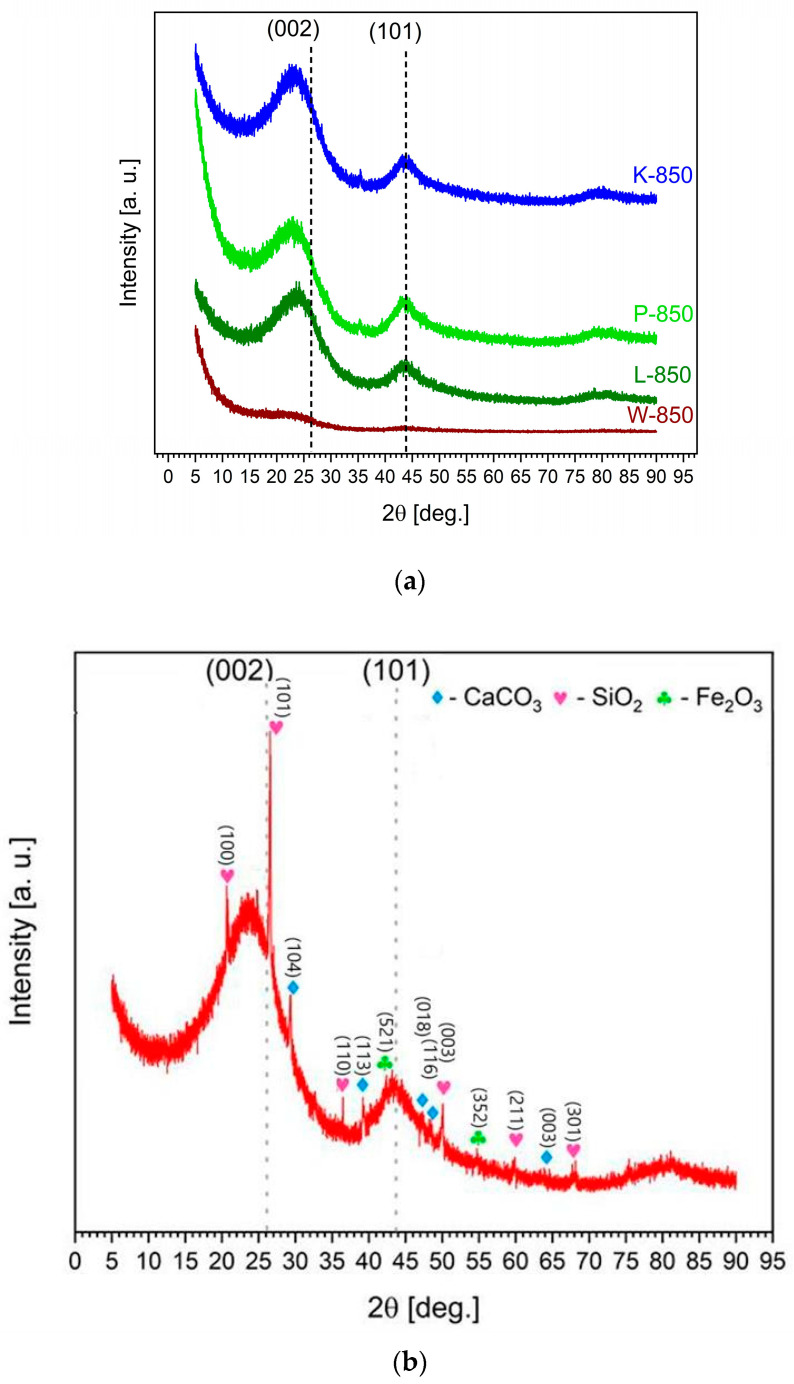
(**a**) X-ray diffraction pattern recorded for biochars W-850, L-850, K-850 and P-850. (**b**) X-ray diffraction pattern recorded for biochar F-850.

Based on the XRD pattern shown in Figure 2a, the monophase carbon materials obtained exhibited a low degree of ordering in their crystal structure. In the range of low 2θ values, a high background level typical of carbon materials and synthetic carbons with a low degree of ordering was observed.

The XRD pattern in Figure 2a shows reflections at (002) and (101), which are characteristic of graphite planes, indicating the presence of carbon particles with a partially ordered structure. Widened reflections of very low intensity, visible at angles of 25° and 45°, suggest the presence of a small fraction of extremely fine crystallites of turbostratic carbons or defective graphite.

The recorded XRD patterns for charred nutshells are in good agreement with existing literature data [39,40].

Figure 2b shows the XRD pattern of the biochar sample F-850, obtained from peanut shells. Here, inorganic phases were detected alongside low-crystallite carbon phases. The detected reflections in the range of 2θ ≈ 24° and 28° were predominantly associated with crystalline phases of calcite (CaCO_3_) and sylvite (KCl). Peaks within the 50–75° range suggested the presence of silicates and quartz associated with magnesium (Mg), calcium (Ca) and manganese (Mn) [41]. Traces of inorganic phases involving Fe_3_O_4_ or other iron (Fe) forms were also detected at a two-theta value of 42.5° in the recorded XRD pattern.

**Figure 3:**
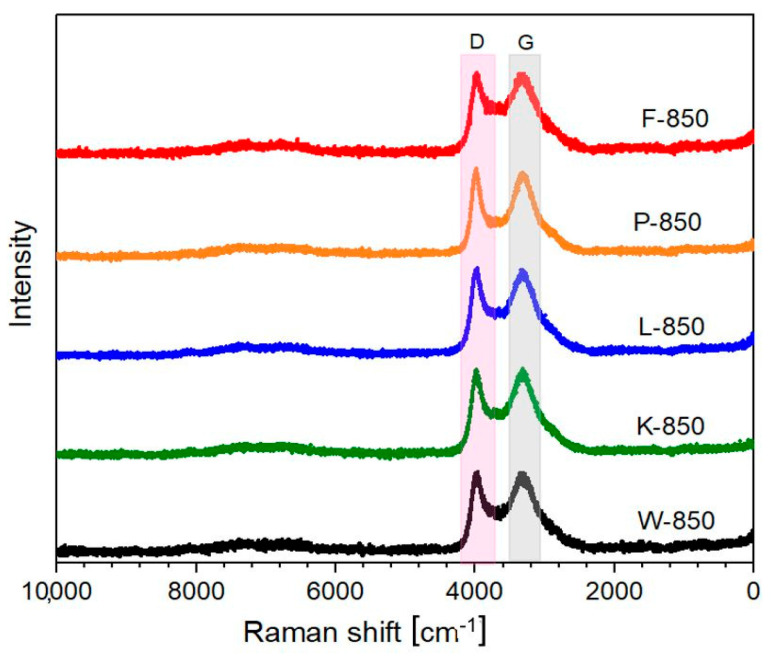
Raman spectra recorded for all investigated biochar samples.

Raman measurements revealed that all investigated samples exhibited typical spectral features of carbon materials, including the so-called D and G bands in the range of 1300–1600 cm^−1^, along with very weak combination bands (D + D and D + G) between 2500 and 3200 cm^−1^ [42,43,44]. The recorded Raman spectra are presented in Figure 3. This process was conducted in the range of 1000–1800 cm^−1^.

**Figure 4:**
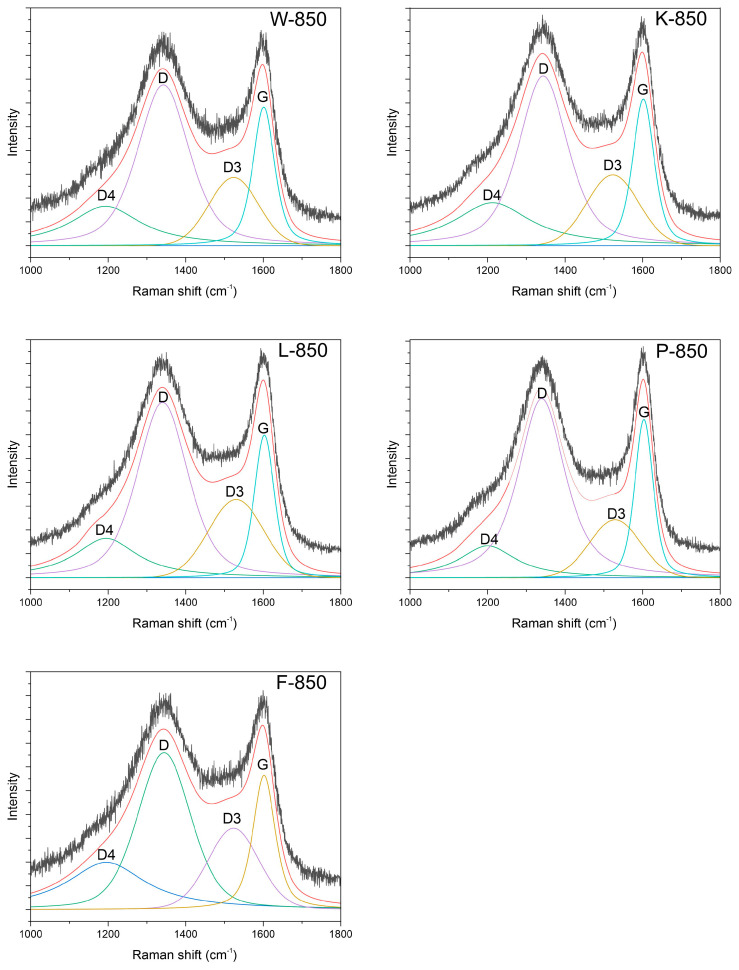
Deconvoluted spectra of all studied biochar samples.

All spectra appeared almost identical, and to perform structural analysis, it was necessary to decompose them into component bands. All recorded spectra were deconvoluted into four component bands, as shown in Table 4 and Figure 4.

These bands included the G band at approximately 1340 cm^−1^, the D band at 1600 cm^−1^, the D3 band at 1520 cm^−1^ and the D4 band at 1200 cm^−1^. The first two can be linked to the E_2_g symmetry in-plane stretching LO phonon vibrations of the Brillouin zone, with the latter corresponding to the A_1_g breathing mode of hexagonal rings [45]. The origin of the D3 and D4 vibrations remains a subject of debate. The D3 band is most often attributed to the amorphous carbon phase or a certain finite graphite phase, whereas the D4 band is linked to the sp^3^ phase in disordered carbons or defects in the carbon structure [46].

The parameters of the individual bands obtained from the deconvolution process, as shown in Table 4, allowed for the calculation of the I_D_/I_G_ ratio.

As indicated, the values for the tested materials were highly similar, ranging between 1 and 2. This suggests that all samples exhibited an almost identical structure. However, the D3 and D4 bands showed distinct variations. The intensities and integrals for both bands varied across samples.

Assuming that the D3 band corresponds to the amorphous carbon phase, the ratio of the intensity of the D3–G bands can be used to determine the amorphous-to-crystalline phase ratio [47,48]. This calculation clearly demonstrated variations in the amorphous phase content depending on the sample, with the lowest value observed for the P-850 sample.

Fourier transform infrared spectroscopy-attenuated total reflectance (FTIR-ATR) in the MID-IR range indicated the non-organic nature of the analysed samples.

**Figure 5:**
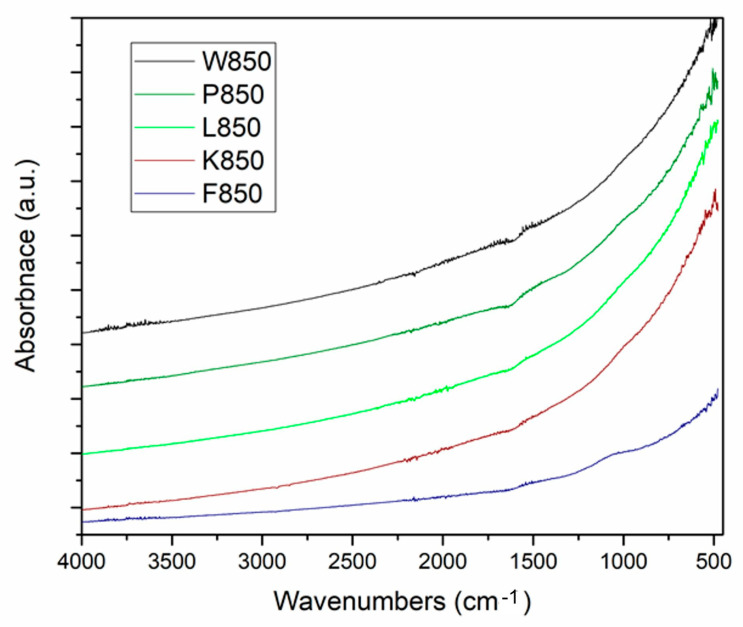
Fourier transform infrared spectroscopy-attenuated total reflectance spectra recorded for biochar samples W-850, P-850, L-850 and F-850.

Figure 5 presents the recorded FTIR-ATR spectra for the investigated biochar samples.

The analysis of the spectra confirmed the non-organic nature of the samples. As expected, the spectra exhibited a shape typical of highly carbonised solid pyrolysis products. In the MID-IR range, no vibration bands of organic compound molecules were recorded, including functional groups typical of aliphatic and aromatic hydrocarbons. The spectrum also lacked carbon–oxygen bonds, both saturated and unsaturated. The baseline was significantly altered at low wavenumbers, indicating an advanced degree of thermal carbonisation in the analysed biochar samples.

Based on the phase composition investigations performed via X-ray analysis and structural tests, it can be concluded that single-phase biochar samples exhibit favourable properties for use as reference biochars in electrochemical studies of the kinetics and mechanism of anodic oxidation in DC-SOFCs.

**Figure 6:**
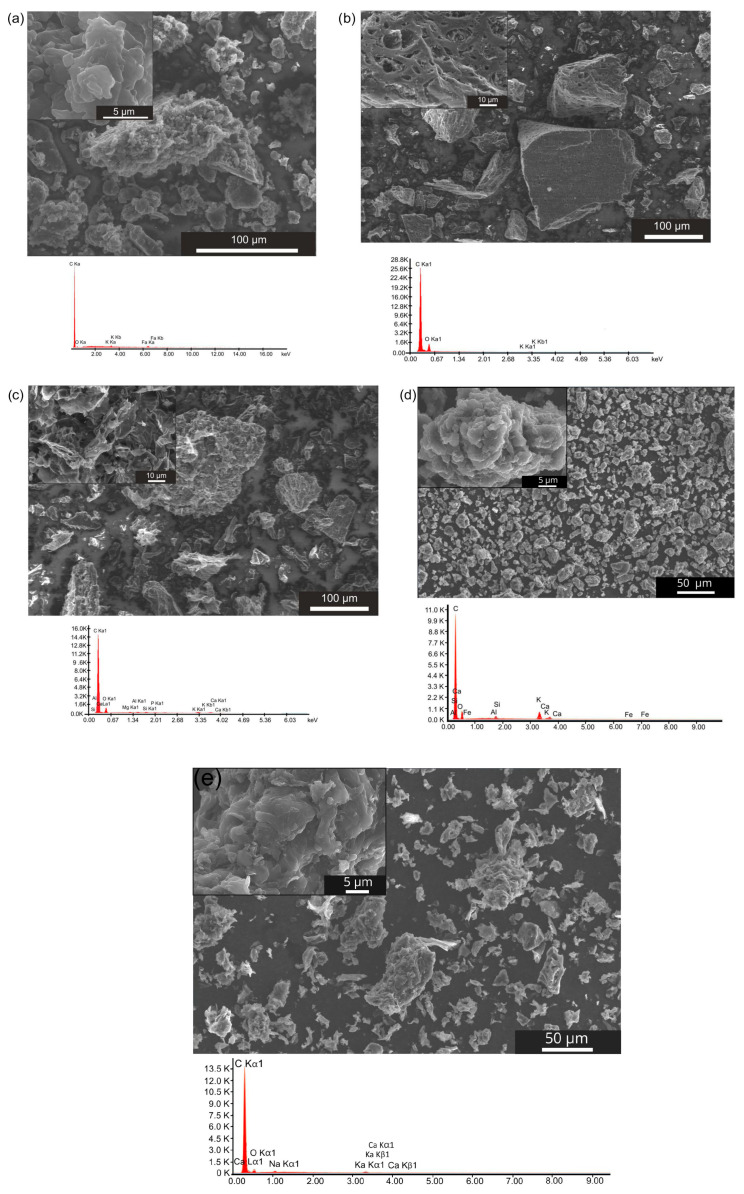
(**a**–**d**) Scanning electron microscopy images of biochars K-850, L-850, F-850 and W-850, including chemical analysis via energy-dispersive X-ray spectroscopy. (**e**) Scanning electron microscopy image recorded for P-850 biochar sample.

The morphological structure of biochar powders, including the shapes of carbon particles and their size distribution, also influences the kinetics and mechanism of electrochemical reactions occurring in the anode chamber of the DC-SOFC. The recorded digital SEM images for all biochar samples are presented in Figure 6a–e.

In Figure 6a, the representative SEM image, coupled with EDX analysis, presents the morphological features of carbon powder obtained from coconut shells (K-850). Figure 6a shows the biochar powder consisted mainly of isometric particles, with sizes ranging from 2 to 65 μm. The EDX chemical analysis indicated the presence of carbon with traces of oxygen adsorbed on the biochar surface. Traces of inorganic matter, such as aluminium (Al), potassium (K), Ca and Fe, were also recorded in the EDX analysis.

The SEM image of carbon particles derived from hazelnut shells is presented in Figure 6b. Based on observations of the morphological structure of the L-850 biochar powder, the powder contained not only isometric particles but also particles of various sizes—some with elongated edges along the y-axis and others with different dimensions along the x-, y- and z-axes.

Other visible differences included an extended particle surface, the presence of sharp edges and single needle-like particles. Moreover, many carbon particles exhibited pores on their surface. The particle sizes of biochar L-850 varied from approximately 2 to 164 μm.

The chemical composition analysis of the L-850 sample indicated that it was characterised by a high carbon content, with a relatively small amount of oxygen present. EDX analysis identified K, Mg and Ca as the main elements in the inorganic compounds.

Based on research on the efficiency of obtaining biochars from hazelnut shells, Toledo et al. [49] found that an increase in process temperature and holding time leads to the formation of a greater number of irregularly shaped grains, as well as an increase in porosity and surface area of the powders. This effect is attributed to the decomposition of lignocellulose components in the samples. The increase in pores and rough surface zones could contribute to a higher specific surface area of the biochar.

Figure 6c shows an image of the morphological structure of F-850 biochar particles. Based on observations and analyses of micrographs recorded under a scanning electron microscope, the powdered biochar F-850 was characterised by a wide range of particle sizes, ranging from 2.5 to 155 μm.

The investigated biochar was characterised by large-shaped particles, with the share of non-isometric particles being much smaller than in L-850. Carbon particles with sharp edges predominated, resembling stone blades.

Based on the chemical composition analysis shown in Figure 6c, the sample contained alkali metal compounds, including K, Ca and Mg. During the analyses, the presence of sulphur in the investigated carbon powder was also confirmed. These results are in good agreement with those reported in [50,51], which investigated biochar powder morphology along with EDX analysis.

The SEM image of biochar W-850 is presented in Figure 6d. Based on observations, it can be concluded that biochar particles obtained from walnut shells are characterised by a somewhat isometric particle shape and an extensive surface resembling fractal geometry. The particle sizes ranged from 3 to 180 μm. The EDX analysis confirmed the presence of K, Ca, Mg, silicon (Si), Al and Fe as components of mineral matter. Figure 6e presents the last analysed sample, P-850 biochar, obtained from pistachio shells.

Cui et al. [52] examined the characteristics of biochar obtained from walnut shells as an adsorbent. Their findings indicated that initial biochar is characterised by a low fraction of porosity. The action of chemical compounds such as KOH leads to an increase in specific surface area and further development of surface structure.

Based on SEM observations, the smallest particle size dispersion was observed in the K-850 biochar sample, followed by the P-850 biochar obtained from pistachio shells. In contrast, the highest particle size dispersion was found in the W-850 and L-850 samples.

The XRF method was also used to verify the chemical composition of the mineral substances integral to the biochar powder.

**Figure 7:**
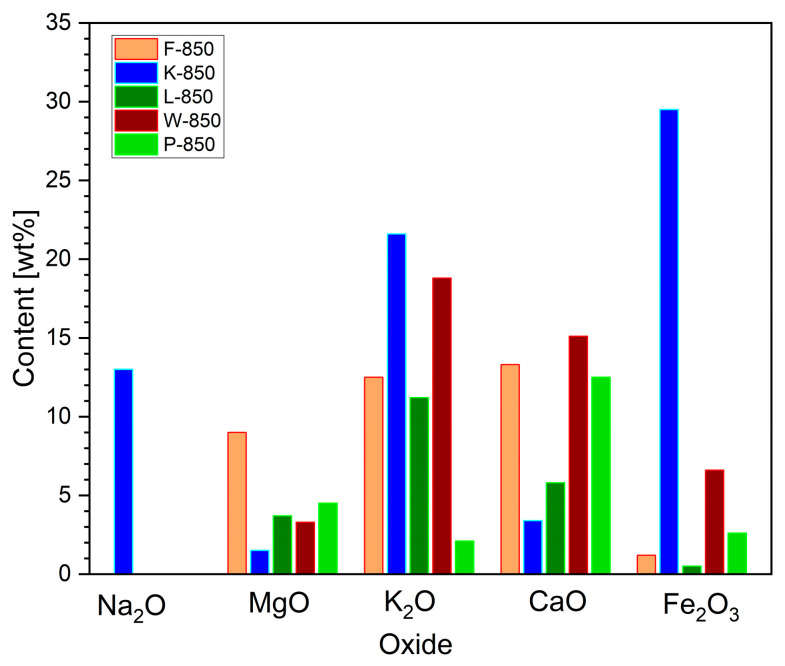
Weight content [%wt] of alkali oxides (Na_2_O, MgO, K_2_O, CaO, Fe_2_O_3_) in biochar samples.

Figure 7 shows the variation in weight content of selected, analysed metal oxides, which are known to be effective catalysts for the Boudouard reaction, a process that can occur in biochar powder.

The XRF analysis indicated that the origin of the feedstock used for biochar production strongly influences the variation in individual oxide compositions.

Here, the K-850 fuel sample had the highest content of Na_2_O, K_2_O and Fe_2_O_3_. High levels of these oxides were also recorded in the W-850 sample. In terms of divalent metal oxides (CaO and MgO), their contents remained relatively consistent across the biochars derived from hard nutshells.

**Figure 8:**
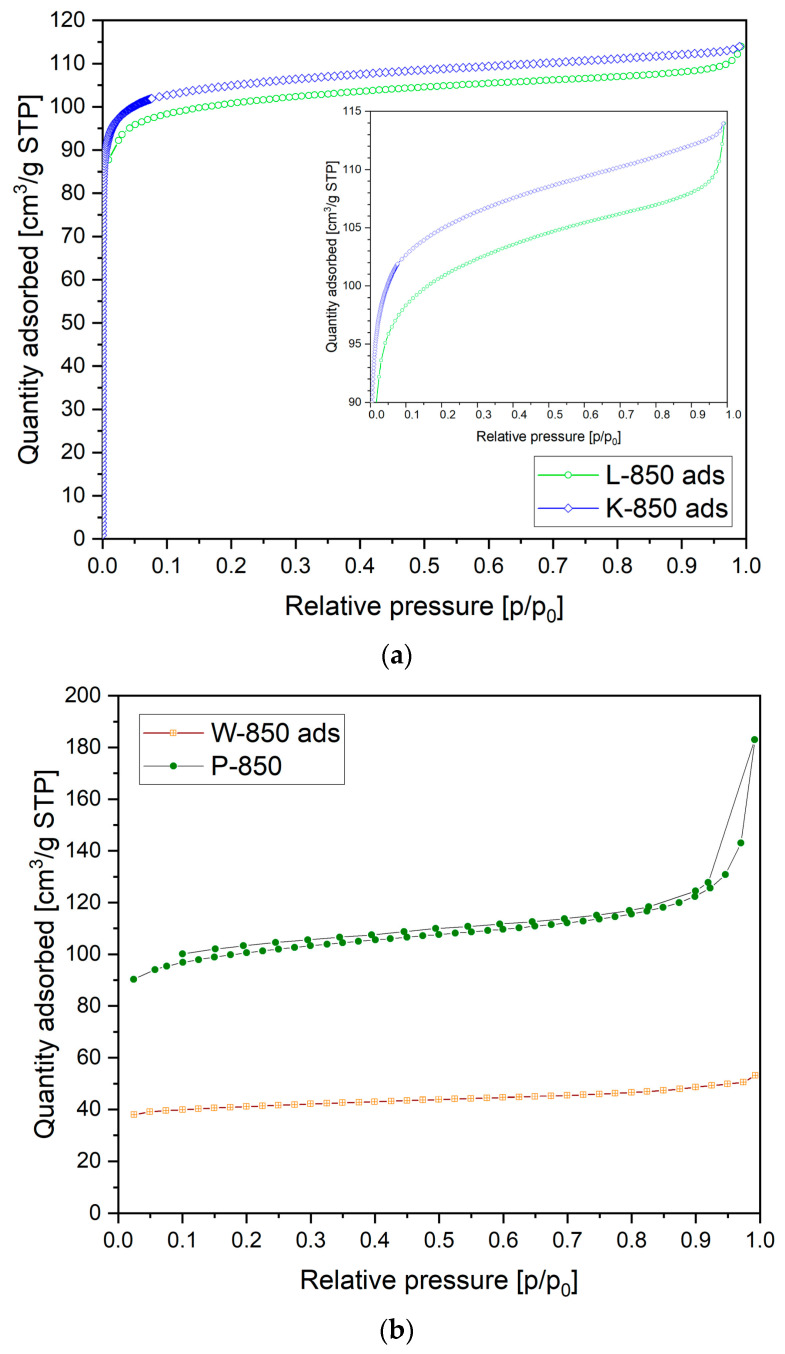
(**a**) Low-temperature nitrogen adsorption/desorption isotherm recorded for L-850 and K-850. (**b**) Low-temperature nitrogen adsorption/desorption isotherm recorded for W-850 and P-850.

The analysis of textural properties of biochar-based materials, including pore size dimensions and their distribution, is also essential for their application as solid fuels in DC-SOFCs. Figure 8a presents the N_2_ adsorption versus relative pressure recorded for the L-850 and K-850 samples.

In Figure 8a, N_2_ adsorption can be observed in the low-pressure range, indicating a significant proportion of microporosity in the carbon materials studied. In the medium- and high-pressure regions, a gradual increase in gas adsorption was visible, suggesting the presence of pores ranging from 2 to 50 nm (so-called mesopores). The isotherm determined for these samples was type IV, which is characteristic of mesoporous materials.

Figure 8b shows the N_2_ adsorption isotherm curve recorded for samples W-850 and P-850. Based on this N_2_ adsorption isotherm, the W-850 and P-850 samples clearly had significantly less developed porosity, both in terms of microporosity and mesoporosity. Table 5 summarises the results reflecting the specific surface area (S_BET_), total porosity volume and the proportion of micropores in the biochar samples.

Based on the data presented in Table 5, it can be concluded that the K-850 biochar sample was characterised by the highest development of the specific surface area (S_BET_). Slightly lower values of specific surface area were obtained for the L-850 and P-850 samples.

The increase in specific surface area is also important for the use of biochar in supplying DC fuel cells. The development of the specific surface area of carbon particles increases the contact area between the particles and the surface of the anode material.

The W-850 sample had the lowest specific surface area and a low proportion of total porosity. The compiled results of the study are in good agreement with the observations made on the morphological structure of individual biochar powders obtained from nutshells. In the case of the L-850 and P-850 samples, intragrain porosity was also visible in the recorded SEM images. XPS analyses were performed to obtain additional information on the atomic composition and electronic properties of the surfaces of the K-850, W-850 and P-850 biochars.

**Figure 9:**
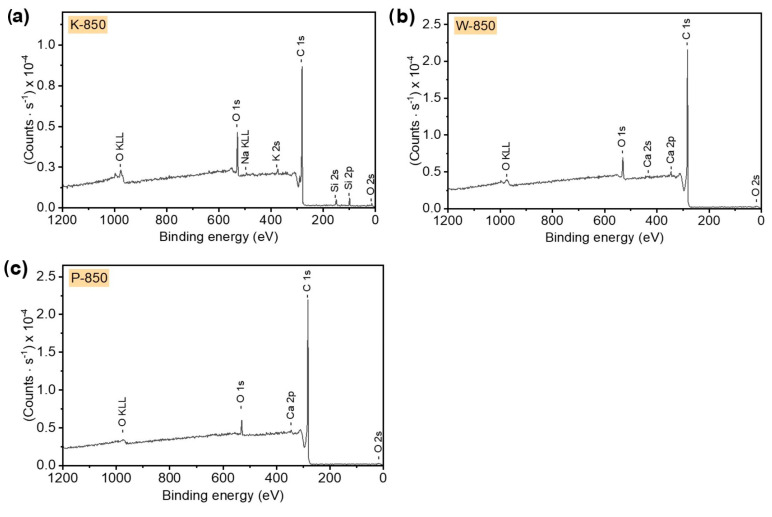
X-ray photoelectron spectroscopy survey spectra recorded for (**a**) coconut, (**b**) walnut and (**c**) pistachio nut biochars.

The recorded survey XPS spectra presented in Figure 9 for the biochar samples obtained from the hard nutshells of coconut (K-850), walnut (W-850) and pistachio (P-850) indicate the high purity of the utilised feedstocks. The high intensity of the C 1s spectra suggests that the obtained charcoals were composed mainly of carbon. The O 1s spectra, of much lower intensity, also indicate the presence of oxygen in all analysed samples. The spectra further reveal trace amounts of sodium (Na), K and Si in K-850; K and Ca in W-850; and Ca in P-850 biochar. Surface concentrations of chemical bonds obtained from fitting XPS data for all analysed samples are listed in Table 6.

**Figure 10:**
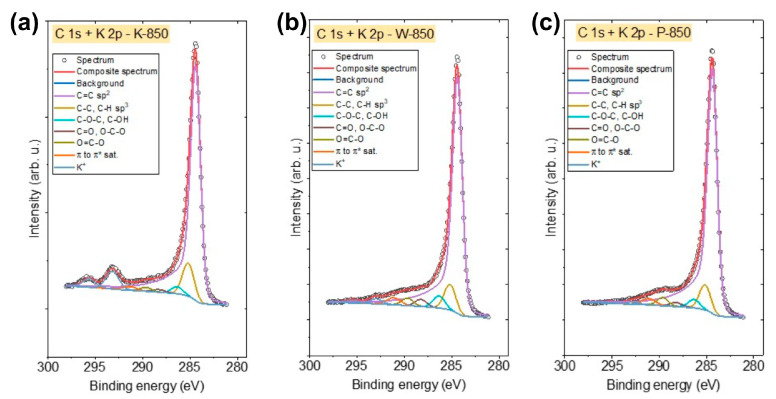
(**a**–**c**) The C 1s and K 2p X-ray photoelectron spectroscopy spectra recorded for (**a**) K-850, (**b**) W-850 and (**c**) P-850 biochars.

**Figure 11:**
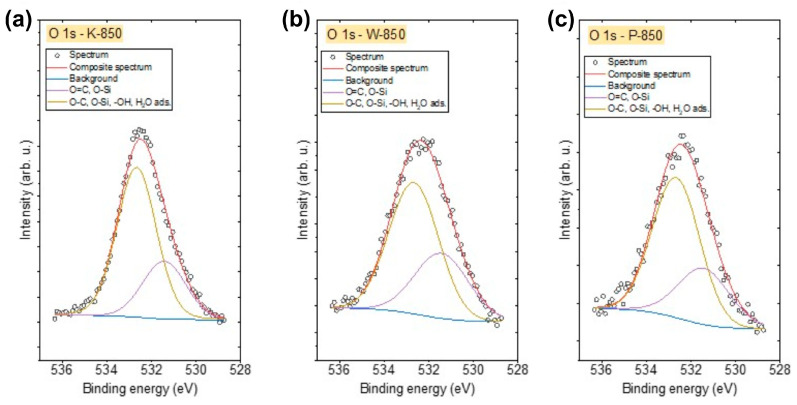
(**a**–**c**) The O 1s X-ray photoelectron spectroscopy spectra recorded for (**a**) K-850, (**b**) W-850 and (**c**) P-850 biochars.

Figure 10a–c and Figure 11a–c show the XPS spectra recorded in the C 1s and O 1s regions. The various components used for the deconvolution of the C 1s and O 1s spectra are also presented in the graphs.

The C 1s spectra of all biochars were fitted with six components. The first peak at 284.4 eV corresponds to graphitic C=C bonds. The second peak at 285.1 eV originates from C–C/C–H groups. The third peak at 286.3 eV corresponds to C–O–C and/or C–OH, while the fourth peak at 288.3 eV indicates the presence of either C=O and/or O–C–O bonds. The fifth peak at 289.4 eV corresponds to O=C–O bonds, while the final peak at 291.1 eV can be attributed to π to π* shake-up satellite. This shake-up excitation originates from sp^2^ carbon and its aromatic forms, serving as an additional parameter confirming the presence of this type of bond [53,54].

The O 1s spectra of all biochars (Figure 11a–c) were similar and were fitted using two components. The first peak, found at 531.4 eV, indicates the presence of O–Si and/or O=C bonds in organic compounds, while the second peak at 532.6 eV corresponds to O–C, O–Si, –OH-type bonds and/or adsorbed H_2_O [55,56]. The highest concentration of oxygen was observed in K-850, whereas the lowest was found in P-850 (Table 6).

**Figure 12:**
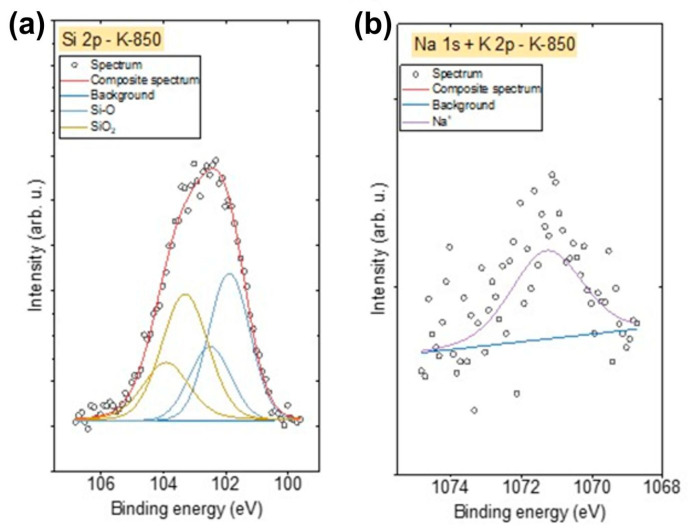
(**a**) Si 2p, Na 1s and (**b**) Na 1s, K 2p spectra recorded for K-850 biochar.

XPS spectra were also recorded in the energy range corresponding to the particular impurities present in the obtained biochars. Due to the presence of Si in the K-850 biochar, Si 2p spectra were also recorded. These spectra were fitted with two doublet structures (Figure 12a) (p_3/2_ − p_1/2_ doublet separation = 0.61 eV), with the main 2p_3/2_ lines centred at 101.9 eV, indicating the presence of Si–O–C bonds, such as in silicone or siloxane species, and at 103.2 eV, corresponding to SiO_2_ [57].

The Na 1s spectrum for the K-850 biochar (Figure 12b) was fitted with one line centred at 1071.3 eV, representing the Na⁺ oxidation state (likely as an oxide), while the K 2p spectrum was fitted with one doublet structure (Figure 12b) (p_3/2_ − p_1/2_ doublet separation = 2.7 eV), with the main 2p_3/2_ line centred at 293.3 eV, indicating the presence of K⁺ ions.

**Figure 13:**
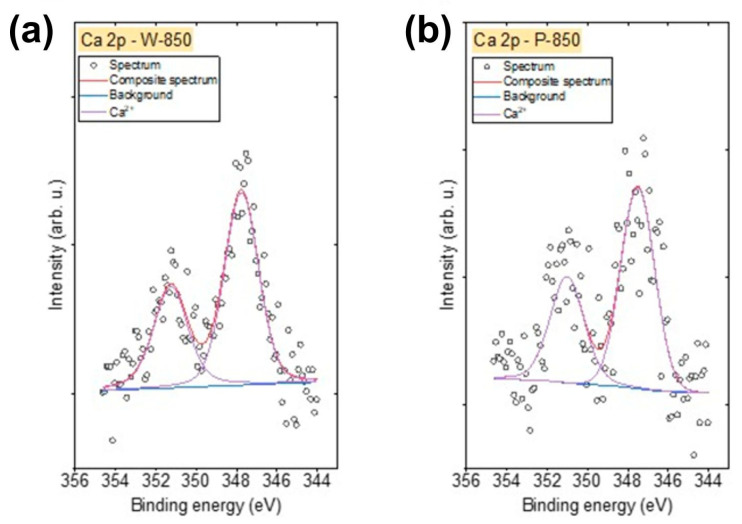
The Ca 2p spectra recorded for (**a**) W-850 and (**b**) P-850 biochars.

Figure 13 shows the spectra Ca 2p spectra recorded for the W-850 and P-850 biochars. The Ca 2p spectra for these biochars were fitted with a doublet structure (p_3/2_ − p_1/2_ doublet separation = 3.51 eV), with the main 2p_3/2_ line centred at 347.6 eV, indicating the presence of Ca^2^⁺ ions in either CaO, Ca(OH)_2_ or carbonates [58].

**Figure 14:**
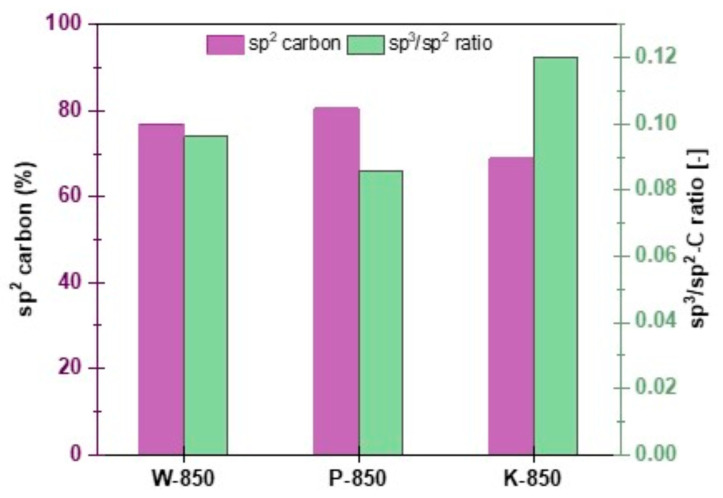
The sp^3^/sp^2^ carbon ratio and sp^2^ phase concentration for W-850, P-850 and K-850 samples estimated via X-ray photoelectron spectroscopy.

Figure 14 presents summarised analysis results for sp^3^/sp^2^ ratio and sp^2^ phase concentration for selected biochars.

XPS quantification of sp^2^- and sp^3^-bonded carbon, as well as oxygen-containing functionalities on the sample surfaces, reflects the quality of the obtained solid carbon residues. As is well known, in typical graphite-like carbon materials, the sp^3^ component represents amorphous, non-crystalline domains and defects present in the carbon structure [59]. Therefore, the amount of sp^2^-bonded carbon can be used to measure the structural integrity and order of sp^2^ carbon forms, while the sp^3^/sp^2^ ratio serves as an indicator of the amount of sp^3^ defects.

The total amount of sp^2^ fractions and the corresponding sp^3^/sp^2^ ratio for the obtained types of carbon residues are shown in Figure 14. Here, the sp^2^ carbon percentage on the sample surface varied from 68.8% to 80.4%, indicating differences in the structural resistance of the samples to annealing. The lowest sp^2^ content was determined for the K-850 sample, which suggests the highest structural disorder of this type of residue and the lowest susceptibility to structural conversion upon annealing. This correlates well with the highest defect ratios. The largest changes towards the planar structure of π-conjugated, defect-free graphite were observed for the P-850 sample, where the concentration of the graphitic sp^2^ phase was the highest after annealing, and the sp^3^/sp^2^ ratio was close to zero.

The surface properties of biochars, mainly their degree of graphitisation, defects and the presence of carbon in individual chemical combinations, determine their chemical and electrochemical reactivity in DC-SOFCs. The presence of oxygen adsorbed on the surface of biochar and the degree of oxidation are additional factors that support the carbon gasification process or the direct oxidation of carbon particles in contact with the surface of the electrolyte or anode material.

### 3.1. Thermal Analysis of Obtained Fully Carbonised Biochar Powders

**Figure 15:**
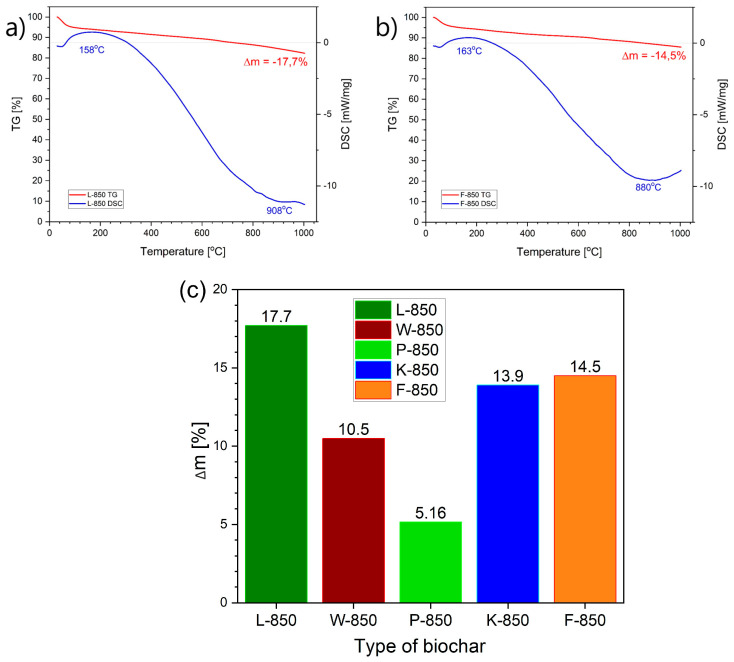
(**a**,**b**) Differential scanning calorimetry (DSC) and thermogravimetric (TG) curves recorded for biochar samples during heating from 20 °C to 1000 °C. (**c**) Dependence of mass loss (∆m) based on thermogravimetric curves for different biochar samples derived from nutshells.

The objective of the comparative study performed using DTA/DSC and TG analysis for L-850, F-850 and K-850 biochar samples was to determine the thermal effects that may occur when these solid fuels are heated during the operation of a DC-SOFC or under DC-SOFC operating conditions in the temperature range of 700–850 °C. The results of DTA/DSC-TG investigations recorded for the W-850 and P-850 samples were previously described in our earlier papers [31,33]. Figure 15a,b presents representative recorded DSC and TG curves for biochar samples obtained from hazelnut shells (L-850 and F-850).

In the DTA/DSC thermal analysis tests performed, no significant thermal effects were observed on the DSC and DTA curves within the temperature range studied. The only visible thermal effects in the DTA curves for biochars obtained from peanut or hazelnut shells were slight peaks at around 153 °C or 163 °C. Further thermal effects were observed only at high temperatures (approx. 880 °C or 908 °C). Slight endothermic peaks at around 153 °C or 163 °C were associated with the evaporation of residual moisture and light organic fractions. Similar correlations or dependencies were recorded for the other biochars, including those derived from walnut or pistachio shells. Figure 15c shows the collected data regarding mass loss (Δm) from TG curves for all biochar samples (W-850, L-850, F-850 and K-850) within the temperature range of 20–1000 °C.

As Figure 15c shows, the highest mass loss was recorded for the biochar sample obtained from hazelnut shells (L-850), followed by samples F-850 and K-850. The lowest mass loss was recorded for biochar obtained from pistachio shells. Based on the recorded variation of mass over the temperature range studied, it can be concluded that the proportion of volatile fractions released from biochar is extremely small.

The above observations allow for a preliminary determination of the possible chemical processes occurring in the carbon bed and the predicted electrochemical reactions taking place in DC-SOFCs. Based on analysis of the literature data and the conducted tests, it can be assumed that the contribution of pyrolytic gases from this family of biochars is negligible and will not significantly impact the achieved values of current density and electrical power output in DC-SOFCs.

**Figure 16:**
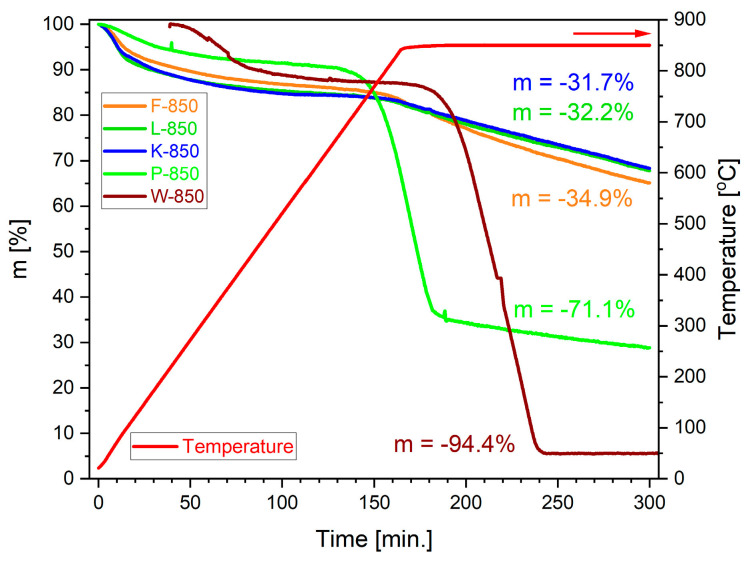
Dependence of m vs. time or temperature recorded under thermogravimetric investigations in a CO_2_ gas atmosphere.

Studying the gasification process of well-formed carbonaceous carbonisation products in a CO_2_ gas atmosphere is another key factor in evaluating the reactivity of biochar in the Boudouard reaction in DC-SOFC fuel cells. Figure 16 presents the collection of mass losses expressed as m versus time.

Up to a temperature of approximately 750 °C, a negligible mass loss of the samples was observed, which was consistent with the TG/DSC results presented in Figure 15a–c. The change in mass within this temperature range is attributed to processes such as moisture evaporation, desorption of adsorbed gases and vapours, and secondary pyrolysis of biochars. The rapid mass loss, observed above 750 °C, is associated with the progression of the Boudouard reaction. Among the analysed biochars, pistachio biochar P-850 exhibited the highest reactivity, which manifested itself in the initiation of the reaction at the lowest temperature and the steepest slope of the TG curve. Raman spectroscopy revealed that biochar samples obtained from pistachio shells showed the lowest degree of graphitisation. A further favourable feature of the pistachio shell samples was their well-developed particle surface (fractal structure), as well as their high porosity. The total volume of pores in the P-850 biochar sample was the highest.

The second most reactive carbonaceous carbonisation product in an atmosphere of CO_2_ was the carbonisation product obtained from the walnut shell biochar (W-850), for which the recorded slope of the TG curve was similar to that of P-850, but the reaction began at a higher temperature. It should be noted, however, that despite the lower reactivity, this material was characterised by the highest degree of conversion. Among the main factors determining the high chemical activity of the W-850 biochar sample were its low degree of graphitisation, high amount of carbon bound to oxygen––which may initiate the surface gasification reaction and high content of various alkali metals, which act as catalysts in the CO_2_ gasification process.

The XRF analyses revealed that sample P-850 was characterised by much lower contents of oxides such as K_2_O or Fe_2_O_3_ but higher proportions of MgO and CaO. Carbonisation products obtained from other shells, namely, hazelnut (L-850), coconut (K-850) and peanut (F-850), exhibited lower reactivity compared to P-850 and W-850. The recorded TG curves for mass loss Δm at set temperatures, as well as over process time, showed similar relationships. The determined conversion degree was approximately 31–35%.

It is noteworthy that the reactivity of this group of biochar is much higher than that of biochar samples made from microcrystalline cellulose (CMK-850) or synthetic carbons such as pyrolytic graphite and carbon black.

### 3.2. Electrochemical Investigations of Performance in DC-SOFCs Supplied by L-850, W-850 and P-850 Biochars

**Figure 17:**
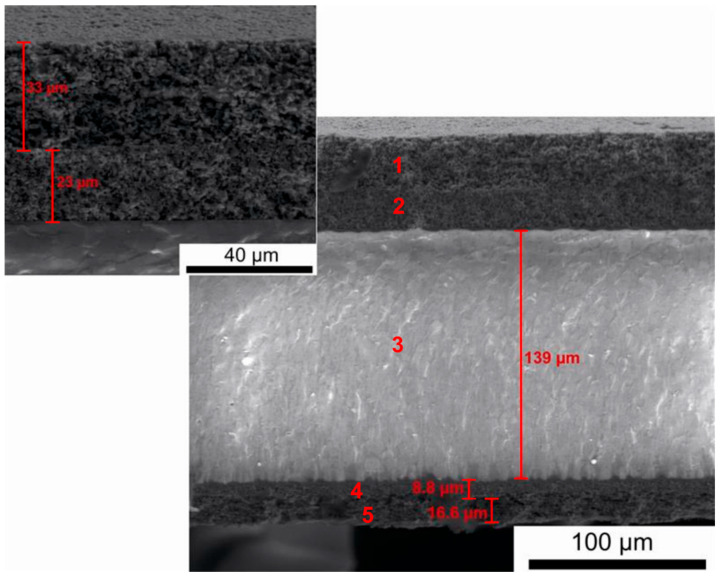
Representative cross-section of an electrolyte-supported solid oxide fuel cell involving an 10Sc1CeSZ electrolyte.

Figure 17 presents a representative cross-section of an electrolyte-supported (E)-SOFC involving a 10Sc1CeSZ electrolyte.

Based on the recorded SEM digital image, the element with the greatest thickness was the gas-tight tape of the 10Sc1CeSZ electrolyte, with a thickness of around 140 μm. The electrode materials had a layered structure with a much smaller total thickness (cathode ≈ 55 μm, anode ≈ 30 μm).

**Figure 18:**
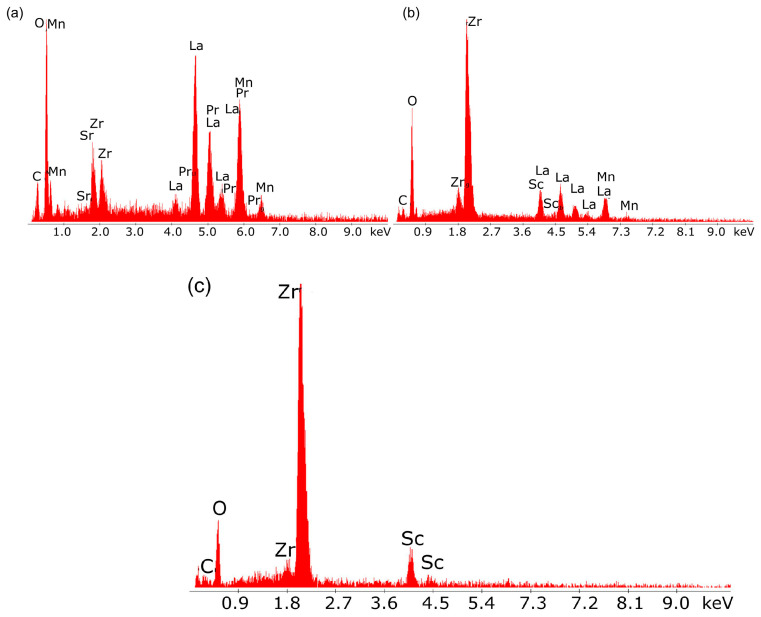
Chemical quality analysis results using energy-dispersive X-ray spectroscopy for (**a**) the cathode (layer no. 1 in Figure 17) and (**b**) the cathode intermediate layer (layer no. 2). (**c**) electrolyte (layer no. 3).

Figure 18a–c shows the results of tests on the chemical composition of the individual layers of the laminate cathode material, electrolyte and laminate anode material.

The results of the qualitative chemical analysis using EDX confirmed the presence of transition metals lanthanum, strontium, and Mn, which form the cathode perovskite material LSM, as well as the presence of scandium (Sc) and Zr in the intermediate layer adjacent to the oxide electrolyte.

**Figure 19:**
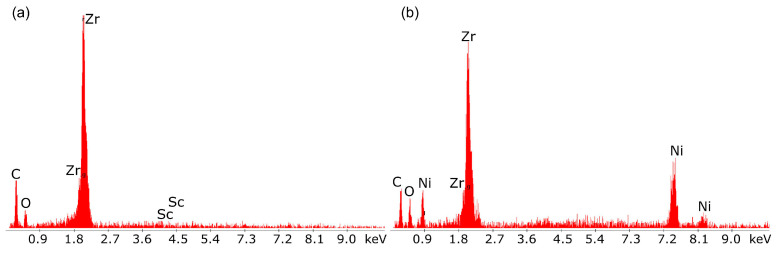
Chemical quality analysis results using energy-dispersive X-ray spectroscopy for (**a**) the cathode (layer no. 4 in Figure 17) and (**b**) the anode intermediate layer (layer no. 5).

Figure 19a,b presents the EDX qualitative chemical analysis results for the oxide electrolyte.

The main components visible in the EDX spectrum are the metals Sc and Zr, which form solid solutions of Sc and Zr oxides, both of which are well-known oxide-ion conductors.

The results of the chemical composition analysis using EDX for the individual components of the layered structure of the porous anode material confirmed the chemical composition of the Ni-ScSZ cermet anode.

**Figure 20:**
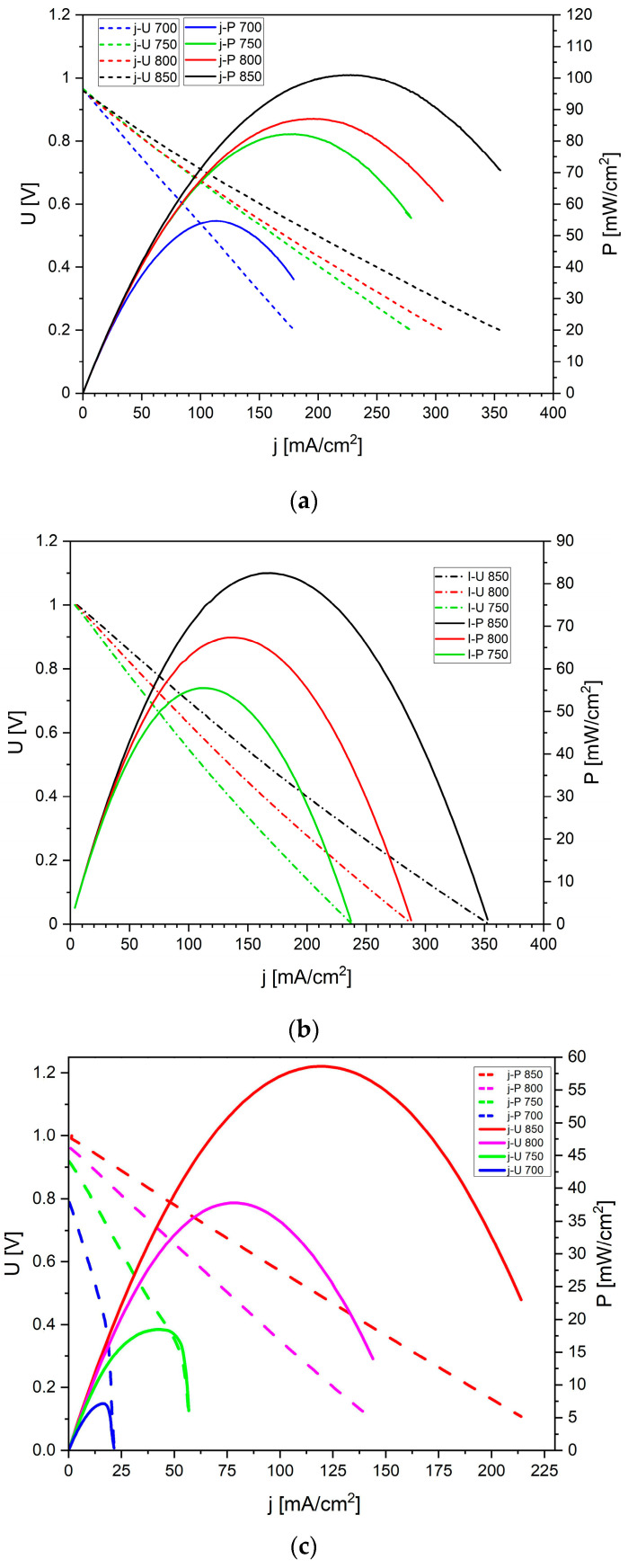
(**a**) Current density (j)–voltage (U) curve recorded for DC-SOFC (I) supplied by biochar W-850. Nitrogen was used as shielding gas. (**b**) Current density (j)–voltage (U) curve recorded for DC-SOFC (I) supplied by biochar K-850. Nitrogen was used as a shielding gas. (**c**) Current density (j)–voltage (U) dependence recorded for DC-SOFC (II) with biochar K-850. Nitrogen was used as a shielding gas.

Figure 20 shows the voltage (*U*) and electrical power (*P*) as a function of current density (*j*) recorded for the DC-SOFCs. Under these experimental conditions, the biochar sample W-850 was used as a solid fuel. An increase in current density and electrical power was observed when the temperature increased from 700 °C to 850 °C. Similar *U-j* and *P-j* dependencies were observed for DC-SOFC (I) and DC-SOFC (II) with biochar samples derived from different nutshells.

Figure 20b presents the *U*-*j* relationship recorded for the same DC-SOFC (I) but supplied with K-850 biochar. A direct comparison of the recorded current density and electrical power for this DC-SOFC exhibited a slightly lower power output compared to DC-SOFC (I) fuelled by the W-850 biochar sample.

Figure 20c illustrates the same functional dependencies recorded for DC-SOFC (II), also fuelled by the K-850 biochar sample. A direct comparison of the operating parameters of DC-SOFC (I) and DC-SOFC (II), both fuelled by the same carbon fuel, indicates that better performance can be achieved when a solid solution of Sc_2_O_3_-ZrO_2_ is used as the electrolyte instead of Y_2_O_3_-ZrO_2_. The impact of differences in the chemical composition of oxide electrolytes or anode materials is reflected in the variations in electrical power derived from DC-SOFC (I) and (II).

**Figure 21:**
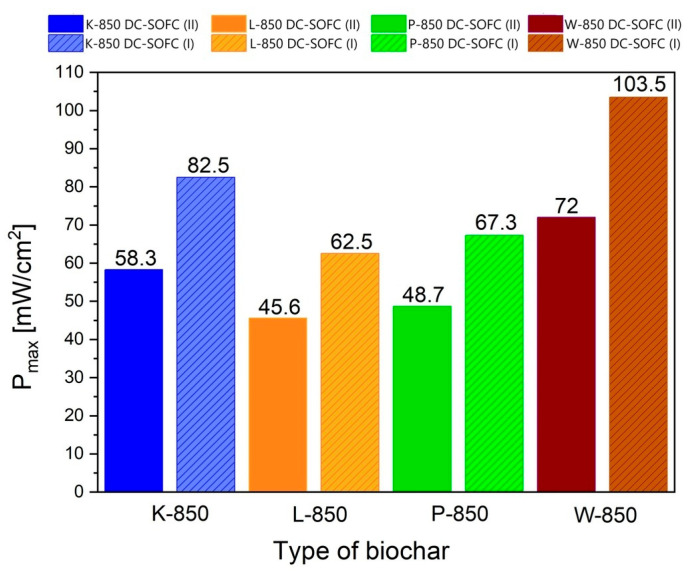
Comparison of performance between DC-SOFC (I) and DC-SOFC (II) supplied by different solid fuels.

Figure 21 shows the direct comparison of electrochemical power output (P_max_) of DC-SOFC (I) and DC-SOFC (II) registered at a temperature of 850 °C.

The results shown in Figure 21 clearly show that the highest current densities and electrical power were achieved for DC-SOFCs (I) and (II) that used the biochar samples W-850 and K-850. The main reason for the better electrochemical performance of the DC-SOFC (I) compared to the DC-SOFC (II), which is operated with the same solid fuel, is the different electrical resistance of the solid electrolyte used. The lower electrical resistance (i.e., higher ionic conductivity) of the 10Sc1CeSZ electrolyte contributes to higher current densities. In addition, the available data from electrochemical tests on SOFC cells fuelled with hydrocarbon fuels indicate that anode materials containing a small amount of cerium (IV) oxide have a higher reactivity in the CO oxidation reaction than the Ni–YSZ anode [60,61,62].

The use of P-850, L-850 and F-850 as solid fuels resulted in a slightly lower electrical output of the DC-SOFCs. The experimental data shown in Figure 21 reflect the influence of the physiochemical properties of the biochar on the performance of the DC-SOFC. In the case of using the W-850 biochar, its superior properties as a fuel can be attributed to the high degree of conversion from the solid carbon phase to single-phase CO. The K-850 biochar powder was characterised by a limited degree of conversion from carbon X to the gas phase CO, but had a relatively high electrochemical reactivity in the DC-SOFC. The K-850 biochar had a significantly higher volume of total porosity in the carbon bed and better development of the specific surface area. Another decisive property was the surface chemistry of the biochar used.

The K-850 sample had the highest content of sp^3^ fractions, which correspond to disordered, defective particles. Morphological characteristics such as a uniform distribution of particle size and preferred isometric shape of the particles are also required to establish a good physical contact between the carbon particles and Ni-YSZ surface properties. The essential difficulty in electrochemical oxidation of solid particles according to reaction (1) is the limited electrode reaction zone for direct contact between the carbon in the solid phase and the electrolyte surface. Based on this finding, it can be concluded that the increase in surface development, as well as the narrow particle size distribution, isometric grain particle shape (i.e., lack of large dimensional differentiation, x, y, z) of the carbon powder, will be factors favourable to the electrochemical oxidation process according to reaction (1). In [63,64], it was demonstrated that the presence of elongated needle-shaped or pyramid-shaped grains in the carbon powder will lead to a further increase in the electrical resistance at the interface of the solid fuel-surface of the electrolytic and the anode material. Among the tested series of powders, the most favourable features are most likely to be found in biochars produced from walnut or coconut shells. According to reactions (2) and (3), the electrochemical oxidation process of coal can occur gradually in the first stage as the oxidation of solid coal to carbon monoxide (II) (reaction [2]) and then of carbon monoxide (II) to carbon monoxide (IV). It should be emphasised that the electrochemical oxidation process of carbon fuel CO is crucial for obtaining high current densities and electrical power output from DC-SOFCs. On the other hand, in [63], the authors indicated that concentration polarisation resulting from insufficient CO concentration during the operation of the DC-SOFC is the main reason for the decrease in the efficiency of the cell. According to descriptions of the shuttle mechanism in the literature, the most important driving force for electrochemical oxidation is CO concentration, which is produced in situ in the carbon bed.

The CO_2_ gasification medium comes from different sources in the anode chamber: (i) the product of electrochemical reactions (1)–(3); (ii) the product of decomposition carbonates, as well as residual decomposition of organic compounds; and (iii) the different process of carbon oxidation. The higher content of alkali metals as Na_2_O, K_2_O and Fe_2_O_3_ also improve the gasification of solid carbon to CO in the carbon bed.

It is also well known that CO (g) formation is thermodynamically favoured at high temperatures, particularly in the 800–850 °C range. This phenomenon directly reflects the thermodynamics of the Boudouard equilibrium. The better performance of DC-SOFC (I) and (II) supplied by biochars using walnut shells and coconut shells can be explained through the shuttle mechanism of DC-SOFC performance. In the case of biochar W-850, the driving force was CO produced in the carbon bed during the conversion of solid phase to gas phase in CO_2_ gas atmosphere. In the case of K-850, the surface properties detected via XPS and the presence of alkali metals were crucial for good performance. Furthermore, the K-850 sample possessed good textural properties in terms of the development of specific surface area and high microporosity content in the carbon bed. In this case, these specific surface properties were responsible for sufficient CO production rate in the anode chamber. When the CO gas production or transport rate could not support the DC-SOFC anode operating in a large current density discharge, a decrease in the performance of this sample was observed. It has been reported that the kinetic limitations of CO gas transport in a porous medium such as a formed carbon bed may have a decisive influence on the kinetics of the electrochemical CO oxidation process in a DC-SOFC [65].

The use of the remaining biochars as solid fuels to power DC-SOFCs led to achieving moderate operating parameters in the range of around 50–60 mW/cm^2^, depending on the type of DC-SOFC and the type of solid fuel.

**Figure 22:**
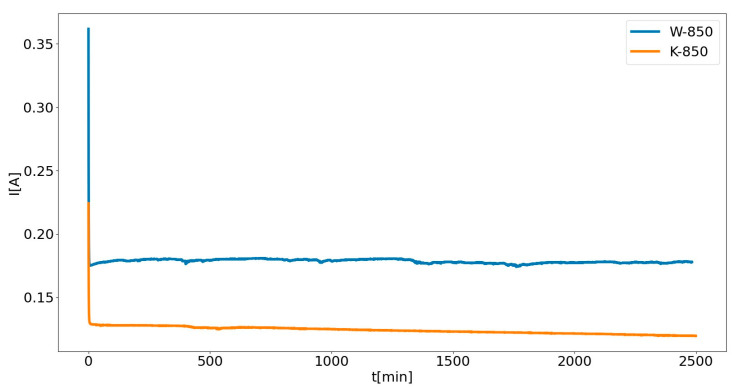
Chronoamperometry curve current, I vs. time recorded under polarization U = 0.5 V for DC-SOFC (II) using biochars W-850 or K-850 as solid fuels.

Figure 22 presents the electrochemical performance of DC-SOFC (II) using the W-850 or K-850 biochars under constant electrical load U = 0.5.

**Figure 23:**
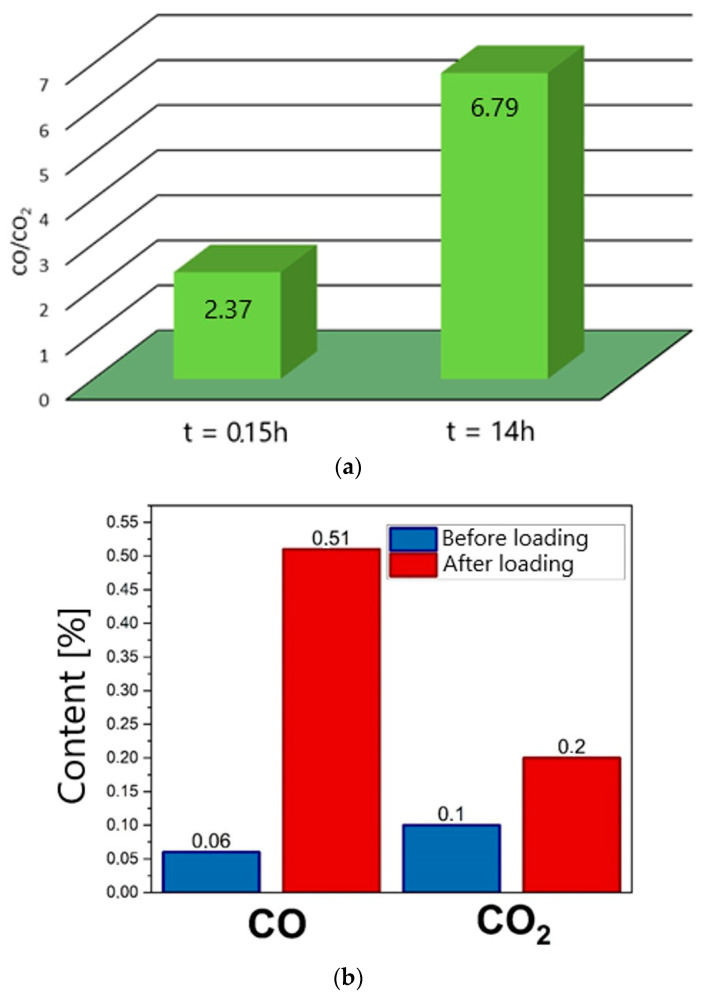
(**a**) Variation in CO/CO_2_ ratio determined for biochar W-850 placed in the anode chamber of DC-SOFC (II). (**b**) Variation in CO and CO_2_ content in outlet gases from the anode before electrical load or after electrical load.

The observed increase in current density (*j*) over time (*t*) is related to the formation of CO, possibly as a result of a Boudouard reaction (C + CO_2_ = CO) occurring in the carbon bed. The first factor responsible for the increase in CO concentration and the subsequent increase in current density were the changes occurring in the bed, related to the gasification of solid phase. This is confirmed by the results presented in Figure 23a, which shows changes in the CO/CO_2_ ratio determined at different time intervals for a W-850 biochar sample placed in the anode chamber. These changes were determined for DC-SOFC (II), where electrical load *U* = 0.

As Figure 23a shows, there was a gradual increase in the ratio of CO to CO_2_ gas products depending on the time of thermal treatment of biochar in the anode chamber. The DC-SOFC was not loaded and was in steady-state condition (where *U* = 0).

Figure 23b shows the variation in CO and CO_2_ content in the outlet gases determined via chronoamperometry. Measurements were performed for DC-SOFC (II) under the conditions of OCV = 1 V and electrical load *U* = −0.5 V.

The results for the changes in CO or CO_2_ gas product content in the exhaust gases from the anode chamber confirm the importance of carbon oxide (II) as a key reactant responsible for both the operating parameters of DC-SOFCs and the stability of their operation over time. As Figure 23b shows, as a result of the operation of the DC-SOFC under the voltage load U = 0.5 V, there was an increase in the concentration of CO as a gasification product, where the gasification medium was CO_2_. The results shown in Figure 23a,b confirm the importance of CO as the main reagent in the electrochemical anodic oxidation of fuel, which can be described as a shuttle mechanism of DC-SOFC performance

## 4. Conclusions

Biochars from walnut, coconut, hazelnut and pistachio shells appear to be valuable solid fuel cells for DC-SOFCs. The family of investigated char samples is characterised by high carbon content and low mineral matter. The main feature of such solid fuels is naturally accumulated alkaline elements that can act as natural catalysts for the reverse Boudouard reaction in the anode chamber of DC-SOFCs. The X-ray and Raman spectroscopy studies showed that the fully carbonised biocarbon consisted mainly of disordered carbon particles. Analysing the deconvoluted parameters of I_D3_/I_G_ from Raman spectra allowed us to determine the ratio of amorphous to crystalline phases. The lowest I_D3_/I_G_ factor was found for biochar from pistachio shells, followed by biochar from walnut and coconut shells. The SEM investigations enabled us to identify the differences in the morphological structures of the carbon powders. The biochar powders obtained from walnut and coconut shells consisted mainly of isometric particles. These particles in turn formed larger clusters known as powder agglomerates. The structure of these biochar powders resembled a fractal pattern. These morphological characteristics represent positive features for application as solid fuel in DC-SOFC. Based on the described results of the analytical investigations and evaluation physiochemical properties of the carbon-based samples—including the total content of carbon, hydrogen, and sulphur; the obtained ratio between the amorphous and crystalline phases; and other microscopy and textural studies—it can be clearly stated that the samples obtained from walnut and coconut shells had the most favourable properties for use as solid fuels for the operation of DC-SOFCs.

The dependencies of mass loss vs. time studies carried out in CO_2_ gas atmosphere at 850 °C revealed that the biochars obtained from walnut and coconut shells had the highest conversion rate from the solid phase to the gaseous CO phase. The main factors contributing to the high chemical activity of the W-850 biochar sample were its low degree of graphitisation, high amount of carbon bound to oxygen—which can trigger the gasification reaction on the surface—and high content of key alkali elements such as Na, K, Mg, Ca and Fe, which act as catalysts in the CO_2_ gasification process. It was found that the use of solid solutions of Sc_2_O_3_-CeO_2_-ZrO_2_ as components for the construction of DC-SOFCs (i.e., as an oxide electrolyte or cermet component acting as an anode) led to higher current densities and electrical power compared to DC-SOFCs using 8% mol Y_2_O_3_ in ZrO_2_ as an electrolyte and anode material component. The electrochemical investigations of two types of DC-SOFCs showed that, in both cases, the highest electrical power was obtained from cells supplied by biochars derived from walnut shells. The results obtained clearly indicate the crucial role played by the conversion of solid-phase carbon into gas-phase CO––which takes place in situ in the anode chamber of direct carbon-solid oxide fuel cells––in the received maximum electrical power and current density.

## Figures and Tables

**Table 1:** Designations for solid fuels used in this paper.

Item	Source of Solid Fuel	Abbreviated Designations	
		Biochar	Raw Shells
1	Hazelnut shells	L-850	L-RS
2	Coconut shells	K-850	K-RS
4	Walnut shells	W-850	W-RS
5	Pistachio shells	P-850	P-RS
6	Peanut shells	F-850	F-RS

**Table 2:** List of components used to construct DC-SOFCs.

Type	Anode	Electrolyte	Cathode
DC-SOFC (I)	NiO-10Sc1CeSZ (Following reduction: Ni-10Sc1CeSZ)	10Sc1CeSZ (10 mol% Sc_2_O_3_–1 mol% CeO_2_–89 mol% ZrO_2_)	LSM La_0.8_Sr_0.2_MnO_3_ LSM +GDC Composite cathode: LSM + 5 mol% Gd_2_O_3_ in CeO_2_
DC-SOFC (II)	NiO-8YSZ (Following reduction: Ni-8YSZ)	8YSZ (8% mol Y_2_O_3_ in ZrO_2_)	LSM (La_0.8_Sr_0.2_MnO_3_) LSM + GDC Composite cathode: LSM + 5 mol % Gd_2_O_3_ in CeO_2_

**Table 3:** Characteristics of raw shells and biochars used in the study.

Sample	Element			Ash [%]
	Carbon	Hydrogen	Sulphur	
K-RS	40.3	5.21	<0.01	0.8
L-RS	48.6	5.61	0.02	0.3
W-RS	46.8	5.5	<0.01	0.4
P-RS	48.1	6.8	0.03	0.2
F-RS	37.9	4.62	0.09	1.6
K-850	94.5	0.24	<0.01	1.1
L-850	92.7	0.23	0.01	1.3
W-850	95.6	0.24	<0.01	1.8
P-850	88.4	1.18	<0.01	1.4
F-850	86.8	0.50	0.11	5.2

**Table 4:** Deconvoluted parameters for selected biochar samples derived from nutshells.

Sample	Band	Position	Intensity	Integral Intensity	I_D_/I_G_	I_D3_/I_G_
		cm^−1^	cm^−1^	cm^−1^		
W-850	D4	1193	0.33	113.68		
	D	1342	1.35	276.45	1.16	0.49
	D3	1524	0.57	91.61		
	G	1601	1.17	102.20		
K-850	D4	1213	0.36	236.69		
	D	1343	1.43	149.75	1.16	0.48
	D3	1523	0.59	157.93		
	G	1602	1.23	65.59		
L-850	D4	1195	0.33	192.93		
	D	1340	1.47	154.41	1.23	0.55
	D3	1530	0.66	166.96		
	G	1603	1.20	60.98		
P-850	D4	1200	0.27	175.18		
	D	1340	1.51	141.85	1.13	0.37
	D3	1529	0.49	154.52		
	G	1603	1.33	56.09		
F-850	D4	1195	0.40	151.71		
	D	1344	1.32	262.23	1.17	0.61
	D3	1523	0.68	119.54		
	G	1602	1.13	104.61		

**Table 5:** Textural characteristics of the obtained biochars.

Sample	Brunauer–Emmett–Teller Surface Area [m^2^/g]	Pore Volume [cm^3^/g]	Micropore Volume [cm^3^/g]
L-850	307.15	0.177	0.1345
K-850	440.30	0.162	0.1432
W-850	126.63	0.082	0.0523
P-850	310.59	0.284	0.1213

**Table 6:** Surface composition (atomic %) determined by X-ray photoelectron spectroscopy measurements.

Element	Carbon						Oxygen		Potassium	Calcium	Silicon		Sodium
Energy [eV]	284.4	285.1	286.3	288.3	289.4	291.1	531.4	532.6	293.1	347.6	101.9	103.3	1071.3
Species	C=C sp^2^	C-C sp^3^	C-O-C	C=O, O-C-O	O=C-O	π to π* sat.	O=C, O-Si	O-C, O-Si, OH	K^+^	Ca^2+^	SiO	SiO_2_	Na^+^
W-850	76.9	7.4	3.3	1.9	2.0	2.0	2.1	3.7	0.2	0.4	0.0	0.0	0.0
P-850	80.4	6.9	2.6	1.3	1.8	2.4	1.4	2.9	0.0	0.3	0.0	0.0	0.0
K-850	68.8	8.3	1.7	0.7	0.9	1.1	3.3	9.2	1.2	0.0	2.1	2.6	0.2

## Data Availability

The original contributions presented in this study are included in the article. Further inquiries can be directed to the corresponding author.

## Abbreviations

The following abbreviations are used in this manuscript:

SOFCs	Solid oxide fuel cells
DC-SOFCs	Direct carbon solid oxide fuel cells
P_max_	maximum electrical power output
L-850	Biochar derived from Hazelnut shells
K-850	Biochar derived from Coconut shells
W-850	Biochar derived fromWalnut shells
P-850	Biochar derived from Pistachio shells
F-850	Biochar derived from Peanut shells
XRD	X-ray diffraction method
MID-IR	Mid-infrared range
IR	Infrared spectroscopy
XPS	X-ray photoelectron spectroscopy
SEM	Scanning electron microscopy
EDX	Energy-dispersive X-ray spectroscopy
XRF	Wavelength dispersive X-ray fluorescence
DTA	Differential thermal analysis
DSC	Differential scanning calorimetry
TG	Thermogravimetry
FTIR-ATR	Fourier transform infrared spectroscopy-attenuated total reflectance
S_BET_	Specific surface area
Δm	Mass loss
U	Voltage
P	Electrical power
j	Current density
